# A phase ΙI study of five peptides combination with oxaliplatin-based chemotherapy as a first-line therapy for advanced colorectal cancer (FXV study)

**DOI:** 10.1186/1479-5876-12-108

**Published:** 2014-04-30

**Authors:** Shoichi Hazama, Yusuke Nakamura, Hiroaki Tanaka, Kosei Hirakawa, Ko Tahara, Ryoichi Shimizu, Hiroaki Ozasa, Ryuichi Etoh, Fumiaki Sugiura, Kiyotaka Okuno, Takumi Furuya, Taku Nishimura, Koichiro Sakata, Kazuhiko Yoshimatsu, Hiroko Takenouchi, Ryouichi Tsunedomi, Yuka Inoue, Shinsuke Kanekiyo, Yoshitaro Shindo, Nobuaki Suzuki, Shigefumi Yoshino, Hirokazu Shinozaki, Akira Kamiya, Hiroyuki Furukawa, Takeharu Yamanaka, Tomonobu Fujita, Yutaka Kawakami, Masaaki Oka

**Affiliations:** 1 Department of Digestive Surgery and Surgical Oncology, Yamaguchi University Graduate School of Medicine, Ube, Japan; 2 Department of Medicine and Surgery, The University of Chicago, Chicago, IL, USA; 3 Department of Surgical Oncology, Osaka City University Graduate School of Medicine, Osaka, Japan; 4 Department of Surgery, Kure-Kyosai Hospital, Kure, Japan; 5 Department of Surgery, Ogori Daiichi General Hospital, Yamaguchi, Japan; 6 Department of Surgery, Kinki University Faculty of Medicine, Osaka-Sayama, Japan; 7 Department of Surgery, Kanmon-Medical Center, Shimonoseki, Japan; 8 Department of Surgery, Shimonoseki-Kosei Hospital, Shimonoseki, Japan; 9 Tokyo Women’s Medical University Medical Center East, Tokyo, Japan; 10 Department of Pharmacy, Yamaguchi University Hospital, Ube, Japan; 11 Department of Biostatistics, National Cancer Center, Chiba, Japan; 12 Division of Cellular Signaling, Institute for Advanced Medical Research, Keio University School of Medicine, Tokyo, Japan

**Keywords:** Peptide vaccine, Peptide cocktail, Colorectal cancer, Phase II study, FOLFOX, Chemotherapy

## Abstract

**Background:**

We previously conducted a phase I trial for advanced colorectal cancer (CRC) using five HLA-A*2402-restricted peptides, three derived from oncoantigens and two from vascular endothelial growth factor (VEGF) receptors, and confirmed safety and immunological responses. To evaluate clinical benefits of cancer vaccination treatment, we conducted a phase II trial using the same peptides in combination with oxaliplatin-based chemotherapy as a first-line therapy.

**Methods:**

The primary objective of the study was the response rates (RR). Progression free survival (PFS), overall survival (OS), and immunological parameters were evaluated as secondary objective. The planned sample size was more than 40 patients for both HLA2402-matched and -unmatched groups. All patients received a cocktail of five peptides (3 mg each) mixed with 1.5 ml of IFA which was subcutaneously administered weekly for the first 12 weeks followed by biweekly administration. Presence or absence of the HLA-A*2402 genotype were used for classification of patients into two groups.

**Results:**

Between February 2009 and November 2012, ninety-six chemotherapy naïve CRC patients were enrolled under the masking of their HLA-A status. Ninety-three patients received mFOLFOX6 and three received XELOX. Bevacizumab was added in five patients. RR was 62.0% and 60.9% in the HLA-A*2402-matched and -unmatched groups, respectively (p = 0.910). The median OS was 20.7 months in the HLA-A*2402-matched group and 24.0 months in the unmatched group (log-rank, p = 0.489). In subgroup with a neutrophil/lymphocyte ratio (NLR) of < 3.0, patients in the HLA-matched group did not survive significantly longer than those in the unmatched group (log-rank, p = 0.289) but showed a delayed response.

**Conclusions:**

Although no significance was observed for planned statistical efficacy endpoints, a delayed response was observed in subgroup with a NLR of < 3.0. Biomarkers such as NLR might be useful for selecting patients with a better treatment outcome by the vaccination.

**Trial registration:**

Trial registration: UMIN000001791.

## Background

Colorectal cancer (CRC) is the third most common cancer and the second leading cause of cancer-related death in industrialized countries [1]. In the past decade, a combination treatment of fluorinated-pyrimidine with irinotecan (FOLFIRI) or oxaliplatin (FOLFOX, XELOX), with or without monoclonal antibodies such as anti-vascular endothelial growth factor (VEGF) antibody or anti-epidermal growth factor receptor (EGFR) antibody, has markedly improved the prognosis of patients with metastatic CRC (mCRC) [2-6]. However, most of the patients reveal progression of the disease due to chemo-resistance and lose their lives.

As an attempt to validate a new treatment modality to overcome the limited disease control status of mCRC, we conducted a combination treatment of five therapeutic epitope-peptides with chemotherapy. Recent developments in genome-based technologies have enabled us to obtain comprehensive gene expression profiles of malignant cells and compare them with normal cells [7]. We had previously identified three oncoantigens, RNF43 (ring finger protein 43) [8], 34 kDa translocase of the outer mitochondrial membrane (TOMM34) [9], and KOC1 (IMP-3; IGF-II mRNA binding protein 3) [10], as targets for the development of cancer peptide vaccines for CRC.

Although immunotherapy using tumor infiltrating cells (TIL) or vaccine treatment are promising modalities for the treatment of cancer, recent reports have indicated several mechanisms in tumor tissues which make cancer cells escape from immune system attacks [11]. For example, the limited antitumor effects of cytotoxic T lymphocytes (CTL) were explained by tumor heterogeneity; a subset of tumor cells revealed the down-regulation or absence of human leukocyte antigen (HLA) or targeted antigen proteins [12,13]. Since the growth of solid neoplasms is almost always accompanied with neovascularization [14], which is associated with the expression of vascular endothelial growth factor receptor 1 (VEGFR1) [15] and/or VEGFR2 [16], our vaccine treatment also included the peptides derived from VEGFR1 and VEGFR2 that target neovascular endothelial cells. We selected five HLA-A*2402-restricted peptides derived from RNF43, TOMM34, KOC1, VEGFR1, and VEGFR2 for the clinical trial due to the abundance of the HLA-A*2402 allele in the Japanese population (an allelic frequency of approximately 60%) [17]. We previously performed a phase Ι study of a combination vaccine treatment for mCRC, and confirmed the safety and the promising potential of our five-peptide-cocktail treatment to improve the prognosis of advanced CRC [18].

FOLFOX (or XELOX) with/without bevacizumab is a widely-used chemotherapy [4] and has been reported to possibly reduce the number of Tregs [19]. We therefore conducted a phase II study of a cancer vaccine consisting of five peptides in combination with oxaliplatin-based chemotherapy as a first-line therapy for advanced CRC.

The purpose of this study was to evaluate the clinical benefit of this cancer vaccine treatment by adding to oxaliplatin-based chemotherapy. Furthermore, we explored a predictive biomarker for its response and for the selection of patients who are likely to exhibit better treatment outcomes following the vaccine treatment. We here demonstrate a promising result of our combination immuno-chemotherapy and predictive biomarkers for immunotherapy.

### Patients and methods

#### Patients and eligibility criteria

Patients were eligible for enrollment when they were ≥ 20 years old with a histologically confirmed advanced CRC, had one or more measurable lesions according to the Response Evaluation Criteria in Solid Tumors version 1.0 (RECIST), were naïve for chemotherapy, had adequate functions of critical organs, had an ECOG performance status (PS) of 0 or 1, and had a life expectancy of ≥ 3 months. The exclusion criteria were CNS involvement, second primary tumors, active infectious disease, any steroid treatment, or any prior peptide vaccination therapies. Written informed consent was obtained from each patient at the time of enrollment. The study was carried out in accordance with the Helsinki declaration on experimentation on human subjects, was approved by the Institutional Ethics Review Boards of Yamaguchi University (H20-102) and each study site, and was registered in the UMIN Clinical Trials Registry as UMIN000001791.

### Peptides

The RNF43-721 (NSQPVWLCL) [20], TOMM34-299 (KLRQEVKQNL) [9], KOC1(IMP-3)-508 (KTVNELQNL) [21], VEGFR1-1084 (SYGVLLWEI) [22] and VEGFR2-169 (RFVPDGNRI) [23] peptides restricted with HLA-A*2402 were synthesized by American Peptide Company Inc. (Sunnyvale, CA, USA) according to a standard solid-phase synthesis method, and were purified by reverse-phase high performance liquid chromatography (HPLC). The purity (> 95%) and the identity of the peptides were determined by analytical HPLC and mass spectrometry analysis, respectively. Endotoxin levels and the bio-burden of these peptides were tested and determined to be within acceptable levels as Good Manufacturing Practice grade for vaccines.

### Study design

This phase II, single arm, non-randomized, HLA-A status double-blind study was conducted to assess the efficacy of this combination therapy for first-line treatment for advanced CRC. The therapy consisted of a cocktail of five therapeutic epitope-peptides in addition to oxaliplatin-containing chemotherapy. Although the peptides used in this study were HLA-A*2402 restricted peptides, all enrolled patients whose HLA-A status were double-blinded were administrated the same regime of peptide cocktail and oxaliplatin-containing chemotherapy.

The cocktail of 3 mg each of five peptides derived from RNF43-721, TOMM34-299, KOC1-508, VEGFR1-1084 and VEGFR2-169, was mixed with 1.5 ml of incomplete Freund’s adjuvant (IFA) (Montanide ISA51; Seppic, Paris, France) and administered subcutaneously into the thigh or axilla regions on day 1 of each week for 13 weeks, then the vaccination schedule was reduced to once every 2 weeks. Vaccination was continued even if the disease progressed when the patient wished and a primary doctor who provided additional chemotherapies agreed.

Oxaliplatin-containing regimens were administrated concurrently with the vaccination. Detailed informations of the chemotherapies were described in Additional file 1. Briefly, mFOLFOX6 [24,25] consisted of oxaliplatin (85 mg/m^2^) with leucovorin (400 mg/m^2^), followed by a FU (400 mg/m^2^) bolus, and then 2,400 mg/m^2^ continuous infusion with/without bevacizumab (5 mg/kg) [4]. This treatment was repeated every 14 days. XELOX [4] consisted of oxaliplatin (130 mg/m^2^) on day 1 followed by oral capecitabine (1,000 mg/m^2^) twice daily on days 1 through 14 of a 21-day cycle with/without bevacizumab at a dose of 7.5 mg/kg.

### Study objectives

The primary objective was the comparison of the efficacy of the peptide-cocktail plus oxaliplatin-containing regimen on patients with HLA-A*2402 compared with those without HLA-A*2402 by assessing the objective response rate (ORR; complete response (CR) and partial response (PR)). Secondary objectives included comparisons between the two groups for progression free survival (PFS), overall survival (OS), safety, and tolerability. Exploratory end points included the assessments of tumor and blood-based immunological biomarkers.

### Assessments

Medical history, physical examination, chest X-ray, ECG, and carcinoembryonic antigen (CEA) measurements were performed within 21 days before starting the treatment. Assessments of vital signs, ECOG performance status, height, weight, and routine blood analysis (hematology and chemistry) were performed within 7 days of starting the treatment. During treatment, physical examination, hematology, and biochemistry analyses were repeated on day 1 of every treatment cycle. Tumor assessments (computed tomography scan, magnetic resonance imaging) were made before starting the study treatment and were repeated every 4 to 8 weeks after the treatment. The RECIST guidelines were used to define all responses. Signs of hematological toxicity and non-hematological toxicity were assessed according to CTCAE during therapy and for 28 days after the last study drug dose.

### Immunological biomarkers

We investigated the neutrophil/lymphocyte ratio (NLR) and the peripheral blood lymphocyte counts per the entire white blood cells (lymphocyte-%) before the treatment as predictive markers of the efficacy of the vaccination. NLR and lymphocyte-% were determined immediately at each study site.

### Statistical analysis

This study was designed to test the hypothesis that a regime consisting of vaccination plus oxaliplatin-containing chemotherapy is more effective for patients with HLA-A*2402 positive aCRC when compared to those without HLA-A*2402, defining the HLA-A*2402 matched group as the study group and the unmatched group as the control group. Because the response rate of colorectal cancer patients to first line-treatment is generally about 50%, we estimated that a minimum of 40 patients for both arms would be required, assuming a response rate of 50% in the HLA-unmatched control group and 65% in the HLA-matched study group. A two-sided Alpha level of 0.2 and a beta level of 0.5 were assumed.

Response rates were compared by chi-squared test. OS and PFS rates were analyzed by the Kaplan-Meier method and log rank test. For the evaluation of delayed response, we also performed a supplemental analysis of the weighted log-rank tests with the Harrington-Fleming class of weights test for 3 parameter settings (ρ = 0 and γ = 0.5; ρ = 0 and γ = 1; ρ = 0 and γ = 2) [26].

Statistical analyses were performed using SPSS statistics version 20 (SPSS, Chicago, IL, USA) and SAS v9.2. A p value < 0.05 was considered statistically significant.

## Results

### Patients

**Figure 1:**
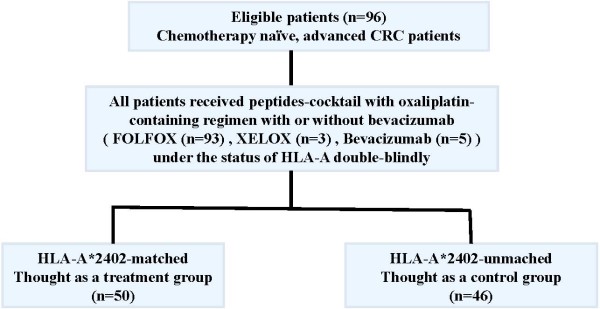
**CONSORT diagram.** Scheme showing an HLA-A-status double-blind, biologically-randomized phase ΙI study of five therapeutic epitope-peptides combined with oxaliplatin-based chemotherapy as a first-line therapy for advanced colorectal cancer (FXV study). CRC, colorectal cancer; FOLFOX, infusional fluorouracil, leucovorin, and oxaliplatin; XELOX, capecitabine and oxaliplatin; HLA, human leukocyte antigen.

Between January 2009 and November 2012, ninety-six patients were enrolled in this trial applying the peptide cocktail treatment in combination with an oxaliplatin-based regimen in 13 hospitals. Fifty patients had at least one allele of HLA-A*2402 and forty-six patients had no HLA-A*2402 allele. The peptide vaccination was administered to all patients. Among the 96 patients enrolled to this trial, 93 patients received mFOLFOX6 and three received XELOX. Five patients were additionally treated with bevacizumab (Figure 1). The baseline characteristics were generally well balanced between the HLA-matched and HLA-unmatched groups, although the proportion of rectal cancer was slightly higher in the HLA-matched group (Table 1). On the cut-off date (25 December, 2013), 87 patients (91%) revealed the progression of the disease with the median OS follow-up period of 38.2 months.

**Table 1:** Baseline Patient Characteristics

**Characteristics**		HLA-A*2402		***p value***
		**Matched (n = 50)**	**Unmatched (n = 46)**	
Sex				
	Male	25	24	NS
	Female	25	22	
Age				
	Mean	64.3	63.4	NS
	Standard error	10.9	8	
	Range	36-82	38-77	
Unresectable site				
	Liver	27	35	
	Lung	18	12	
	Dissemination	5	4	NS
	Bone	1	2	
	Lymphnode	13	13	
	Other	5	1	
Number of unresectable sites				
	1	36	30	
	2	9	11	
	3	5	5	
Resection of primary lesion				
	yes	41	43	
	no	9	3	NS
Chemotherapy				
	FOLFOX	48	45	
	XELOX	2	1	NS
	(Bevacizumab)	0	(5)	
Primary minor site				
	Colon	29	36	0.057
	Rectal	21	10	

FOLFOX. infusional fluorouracil. leucovorin. and oxaliplatin: XELOX. capecitabine and oxaliplatin; HLA, human leukocyte antigen; NS. not significant.

### Objective response rate

The ORR was 62.0% and 60.9% in the HLA-matched and HLA-unmatched groups (p = 0.910), respectively (Table 2). The proportions of CR, PR, and SD as well as the disease control rate were 2.0% (1/50), 60.0% (30/50), 32.0% (16/50), and 94.0% (47/50) in the HLA-matched group, respectively, and 0% (0/46), 60.9% (28/46), 37.0% (17/46), 97.8% (45/46) in the HLA-unmatched group, respectively.

**Table 2:** Objective Response rate

**HLA-status**	HLA-A*2402-matched			**(n = 50)**	HLA-A*2402-unmatched			**(n = 46)**
Response	CR	PR	SD	PD	CR	PR	SD	PD
Number	1	30	16	3	0	28	17	1
Response rate		31/50	(62.0%)			28/46	(60.9%)	

### Progression free survival

**Figure 2:**
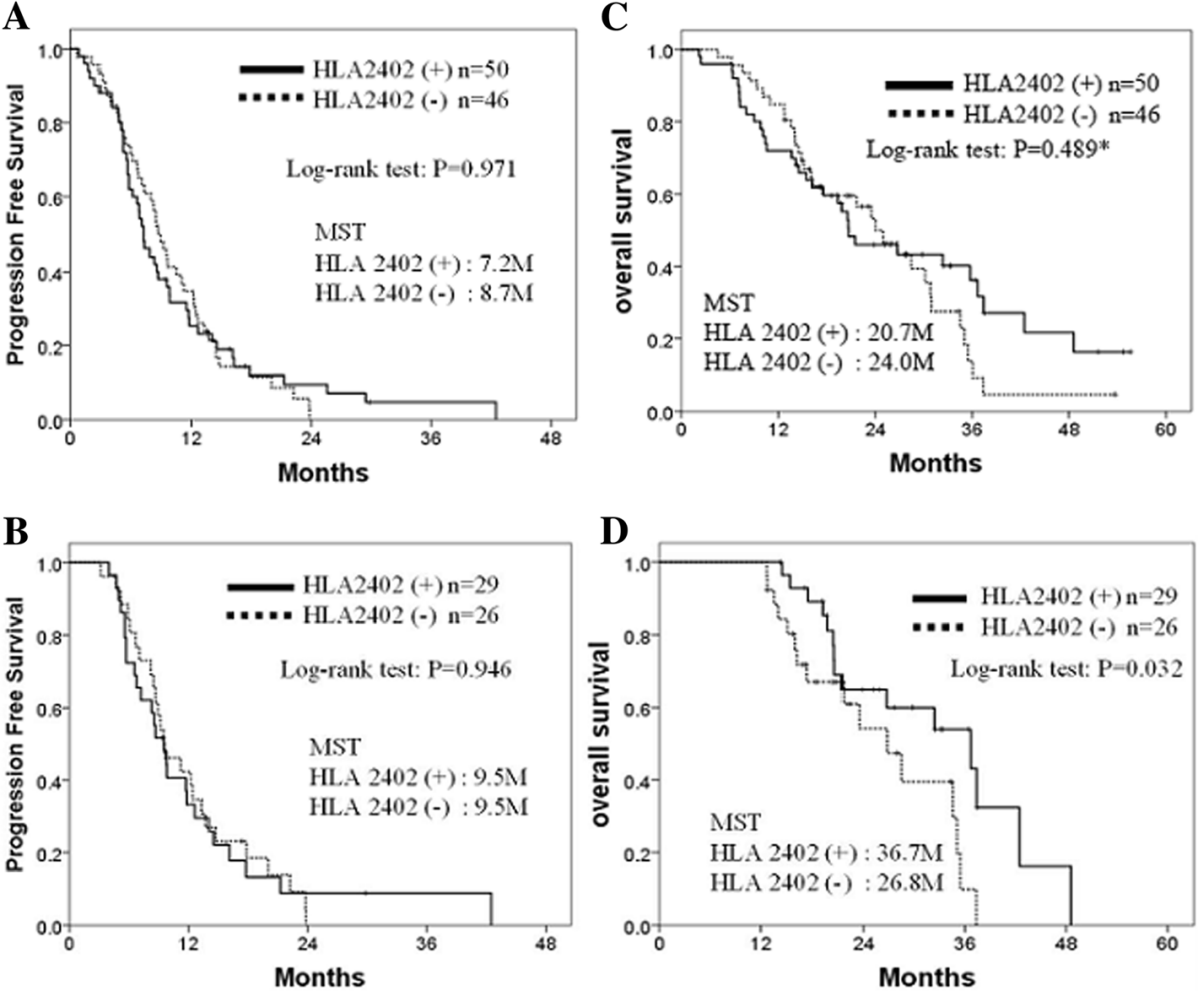
**Progression free survival and overall survival. A** and **B**, comparison of progression free survival between HLA-A*2402-mached and -unmatched groups; **A**, all patients; **B**, the patients who received the vaccination for more than 12 months. **C** and **D**, comparison of overall survival between HLA-A*2402-matched and -unmatched groups; **C**, all patients; **D**, patients who received the vaccination for more than 12 months. MST, median survival time; HLA, human leukocyte antigen; M, months; *the weighted log-rank tests with the Harrington-Fleming class of weights were performed and resulted in, ρ = 0, and γ = 0.5, p = 0.186; ρ = 0, and γ = 1, ρ = 0.080; ρ = 0, and γ = 2, ρ = 0.101.

The median PFS was 7.2 months for the HLA-matched group and 8.7 months for the HLA-unmatched group. There was no significant difference between two groups (Figure 2A, P = 0.971). We also performed sub-group analyses using the patients who received the vaccination for more than 12 months, but there was also no difference between these two groups (Figure 2B, P = 0.946).

### Overall survival

The median OS was calculated to be 20.7 months in the HLA-A*2402-matched group and 24.0 months in the unmatched group. There was no significant difference between the two groups (Figure 2C; log-rank test, p = 0.489; Harrington-Fleming method, ρ = 0 and γ = 0.5, p = 0.186; ρ = 0 and γ = 1, p = 0.080; ρ = 0 and γ = 2, p = 0.101). Interestingly, when the patients were able to receive the vaccination for more than 12 months, the OS of the HLA-A*2402-matched group was significantly better than that of the unmatched group (Figure 2D; log-rank test, p = 0.032).

### Safety

The most common adverse events (AEs) observed in this trial were neurologic toxicity and hematologic toxicities (Table 3). There was no significant difference in the incidence of AEs including injection site reaction in the two groups. Although the incidences of serious adverse events (SAEs) were almost similar in the two groups, that of neutropenia was relatively higher in the HLA-A*2402-matched group than the unmatched group. Interstitial pneumonia that led to the death was observed in two cases in the HLA-matched group and in one case in the HLA-unmatched group (Table 4).

**Table 3:** Frequent and Severe Adverse Events (CTCAE version 3.0)

**FOLFOX (n = 89), FOLFOX + Bev (n = 4), XELOX + Bev (n = 1)**																				
	HLA-A*2402-matched (n = 50)										HLA-A*2402-unmatched (n = 46)									
	**FOLFOX (n = 48), XELOX (n = 2)**										**FOLFOX (n = 41) + Bev (n = 4), XELOX + Bev (n = 1)**									
Adverse Event	1		2		3		4		5		1		2		3		4		5	
	**No**	**%**	**No**	**%**	**No**	**%**	**No**	**%**	**No**	**%**	**No**	**%**	**No**	**%**	**No**	**%**	**No**	**%**	**No**	**%**
Hand-foot syndrome	0	0	0	0	1	2	0	0	0	0	1	2	0	0	1	2	0	0	0	0
Allergy	4	8	3	6	2	4	0	0	0	0	3	7	4	9	0	0	0	0	0	0
Mucositis	2	4	1	2	1	2	0	0	0	0	2	4	0	0	0	0	0	0	0	0
Nausea/vomiting	5	10	1	2	2	4	0	0	0	0	6	13	2	4	1	2	0	0	0	0
Neurologic toxicity	15	30	10	20	4	8	0		0	0	17	37	10	22	5	11	1	2	0	0
Anorexia	10	20	3	6	4	8	0	0	0	0	10	22	4	9	2	4	0	0	0	0
Diarrhea	3	6	6	12	2	4	0	0	0	0	3	7	0	0	1	2	0	0	0	0
Fatigue/Asthenia	5	10	1	2	2	4	0	0	0	0	5	11	1	2	1	2	0	0	0	0
Fever	2	4	0	0	0	0	0	0	0	0	3	7	2	4	0	0	0	0	0	0
Injection site reaction	18	36	18	36	9	18	0	0	0	0	20	43	17	37	3	13	0	0	0	0
Interstitial pneumonia	0	0	0	0	4	8	0	0	2	4	0	0	0	0	4	9	0	0	1	2
Neutropenia	5	10	10	20	10	20	1	2	0	0	8	17	14	30	2	4	1	2	0	0
Leukopenia	10	20	12	24	1	2	0	0	0	0	12	26	9	20	2	4	0	0	0	0
Thrombocytopenia	17	34	3	6	0	0	0	0	0	0	20	43	2	4	0	0	0	0	0	0
Bilirubin	2	4	2	4	0	0	0	0	0	0	0	0	0	0	0	0	0	0	0	0
AL-P	11	22	1	2	1	2	0	0	0	0	10	22	1	2	0	0	0	0	0	0
Creatinine	3	6	1	2	0	0	0	0	0	0	1	2	0	0	0	0	0	0	0	0
Hemoglobin	11	22	5	10	0	0	0	0	0	0	13	28	7	15	0	0	0	0	0	0
Embolism	0	0	0	0	0	0	0	0	0	0	0	0	0	0	0	0	1	2	0	0
AST/ALT	12	24	0	0	1	2	0	0	0	0	6	13	1	2	0	0	0	0	0	0

No gastrointestinal perforation nor bleeding wound healing complication was observed. FOLFOX, infusional fluorouracil, leucovorin, and oxaliplatin; XELOX, capecitabine and oxaliplatin; Bev, bevacizumab; AL-P, alkaline phosphatese; AST, aspartete aminotransfarase; ALT, alanine aminotransferase; CTCAE, the Common Terminology Criteria for Adverse Event version 3.0; HLA, Human leukocyte antigen.

**Table 4:** Interstitial Pneumonia

**HLA**	**CTCAE**	**Result of DLLT**
**genotype**	**grade**	
2402/2402	3	5FU
2402/1101	3	negative
2402/1101	5	negative
2402/0206	3	negative
2402/2603	3	5FU
2402/2602	5	negative
1101/2601	3	5FU
2601/3101	3	5FU
1101/3101	3	5FU
3004/3303	5	not examined
1101/3101	3	not examined

CTCAE, the Common Terminology Criteria for Adverse Event version 3.0; HLA, Human leukocyte antigen; DLTT, drug-induced lymphocyte transformation test; 5FU, 5-fluorouracil.

### Immunological biomarkers

**Figure 3:**
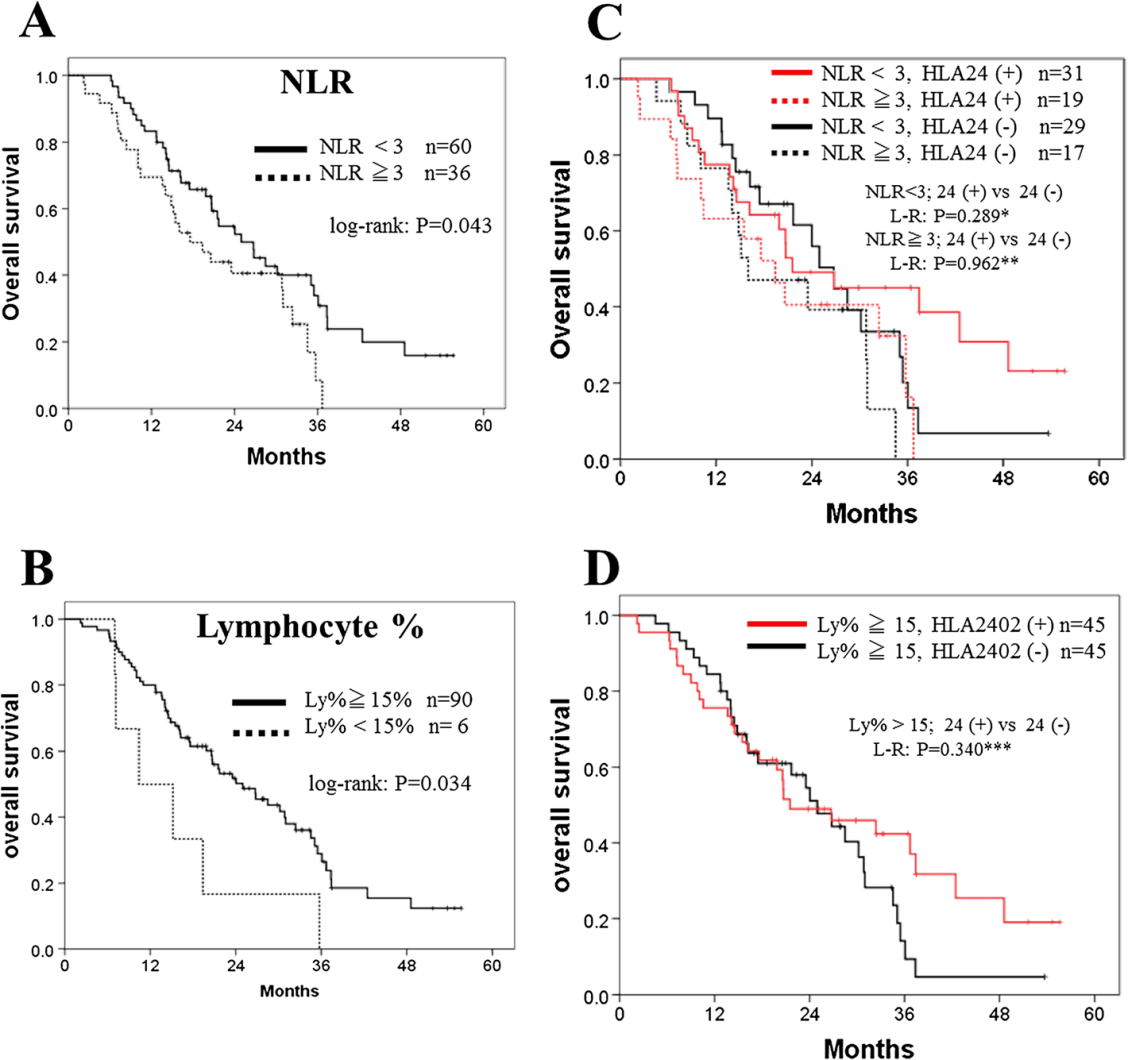
**Biomarkers for the survival and the clinical efficacy of vaccination.** Neutrophil/lymphocyte ratio (NLR) < 3.0 and Lymphocyte-% ≧ 15% were considered as indicative factors. **A** and **B**, comparison between the favorite group and others. **C**, comparison of the patients with a NLR of ≧ 3 or a NLR of <3 between the HLA-A*2402-matched and -unmatched groups. **D**, comparison of the patients with Lymphocyte-% ≧ 15% between the HLA-A*2402 positive and negative groups. Lymphocyte (Ly)-%, the percentage of lymphocytes among the peripheral leukocytes; NLR, neutrophil/lymphocyte ratio; HLA, human leukocyte antigen; L-R, log-rank test; *the weighted log-rank tests with the Harrington-Fleming class of weights were performed and resulted in, ρ = 0, and γ = 0.5, p = 0.152; ρ = 0, and γ = 1, p = 0.064; ρ = 0, and γ = 2, p = 0.035; **the Harrington-Fleming tests were resulted in, ρ = 0, and γ = 0.5, p = 0.495; ρ = 0, and γ = 1, ρ = 0.346; ρ = 0, and γ = 2, ρ = 0.251; *** the Harrington-Fleming tests were resulted in, ρ = 0, and γ = 0.5, p = 0.114; ρ = 0, and γ = 1, ρ = 0.051; ρ = 0, and γ = 2, ρ = 0.029.

NLR is defined as the neutrophil to lymphocyte ratio, and in this study we categorized the patients into two groups (< 3 and ≧ 3) according to the papers reported previously [27]. In this study, NLR of <3.0 was a prognostic marker for the longer survival with peptide cocktail and oxaliplatin-containing chemotherapy (Figure 3A; log-rank test, p = 0.043). The Lymphocyte-% of ≧ 15% was also associated with a long survival (Figure 3B; log-rank test, p = 0.034). Hence, we examined the combined effect of each of these two markers and the HLA types on the clinical efficacy of the vaccination. In patients with a NLR of < 3.0, a significantly longer overall survival was observed in the HLA-A*2402-matched group than the HLA-A*2402-unmatched group (Figure 3C; log-rank test, P = 0.289; Harrington-Fleming method, ρ = 0 and γ = 0.5, p = 0.152; ρ = 0 and γ = 1, p = 0.064; ρ = 0 and γ = 2, p = 0.035) while this difference was not observed in patients with NLR of ≧ 3.0 (log-lank test, p = 0.962; Harrington-Fleming method, ρ = 0 and γ = 0.5, p = 0.495; ρ = 0 and γ = 1, p = 0.346; ρ = 0 and γ = 2, p = 0.251). Similarly, in a patient group with a lymphocyte% of > 15%, a longer overall survival was observed in the HLA-A*2402-matched group (Figure 3D; log-lank test, p = 0.340; Harrington-Fleming method, ρ = 0 and γ = 0.5, p = 0.114; ρ = 0 and γ = 1, p = 0.051; ρ = 0 and γ = 2, p = 0.029).

## Discussion

We performed a phase II study using a cocktail of five epitope peptides, which we previously confirmed its safety, together with oxaliplatin-based chemotherapy. The cocktail contained three peptides derived from three oncoantigens and two peptides targeting VEGFR1 and VEGFR2. This study was an HLA-A-status double-blind, phase ΙI study of five therapeutic epitope-peptides with oxaliplatin-based chemotherapy as a first-line therapy for advanced colorectal cancer (FXV study). In this study, we observed many interesting results.

Firstly, the OS of the HLA-A*2402-matched group was significantly higher compared to that of the unmatched group (log-rank test, p = 0.032) when patients who received the vaccination for more than 12 months (Figure 2D) although no difference in PFS was observed between the two groups (Figures 2B). These results indicated that the additional effect of vaccination on the standard chemotherapy was likely to be slow-acting as this kind of delayed response by the vaccine treatment was indicated in the guidance for therapeutic cancer vaccines released from the US Food and Drug Administration in October, 2011 [28].

Secondly, neutrophil/lymphocyte ratio (NLR) might become a prognostic marker for patients who received the peptide vaccine in combination with standard chemotherapy (Figure 3A, log-rank; p = 0.043), and there was an obvious tail effect for extremely long survival. Then we examined the efficacy of vaccination by comparing HLA-matched group and -unmatched group. In patients with an NLR of < 3.0, a significantly longer survival in the HLA-matched group than the HLA-unmatched group was observed (Figure 3B; log-rank, p = 0.289; Harrington-Fleming, p = 0.035), while this difference was not observed in the two groups with NLR of ≧ 3.0 (log-rank, p = 0.962; Harrington-Fleming, p = 0.251). This result also support the idea that it may be critically important to apply vaccine treatment to patients with better immune status, and NLR might be a one of good predictive markers to select the appropriate patient populations for this type of treatment. A similar result was observed when we analyzed patients with lymphocyte% of ≧ 15%; HLA-matched patients with lymphocyte% of ≧ 15 showed significantly better prognosis than HLA-unmatched patients (Figure 3D; log-rank, p = 0.340; Harrington-Fleming, p = 0.029). The selection of patients with lower NLR and higher lymphocyte percentage might be useful to the selection of patients who are likely to respond well to vaccine treatment and improve clinical outcomes.

Vaccinations with a cocktail of five peptides together with oxaliplatin-based chemotherapy in metastatic CRC patients were well tolerated, except for relatively frequent cases (11 cases; 11.4%) of pneumonitis (Tables 3 and 4), whose incidence seemed to be higher than previously reported for oxaliplatin-based chemotherapies although no difference was observed between HLA-matched and -unmatched group. Correale et al. reported two cases (5.5%) in 36 patients with advanced gastric cancer treated with gemcitabine plus oxaliplatin, folinic acid, and 5-fluorouracil (FOLFOX-4) [29]. Usui et al. reported that four cases (3.9%) of pneumonitis among 104 Japanese patients treated with oxaliplatin-containing regimes for advanced colorectal cancer [30]. In addition, there have been many case reports of oxaliplatin-related pneumonitis [31-35]. In this study, eleven (11.4%) of 96 patients suffered from severe pneumonitis including three cases with grade 5 pneumonitis. To investigate the possible cause of pneumonitiswe performed drug-induced lymphocyte transformation test (DLTT) for nine patients whose samples were available. Among them, five patients (55.6%) were judged to be positive to fluorouracil alone, and the remaining four patients were negative for all of the antigens tested. Although the size of this study is not large enough to make any conclusion and there is no difference between the two groups, this adverse event should be carefully monitored when we will perform the next-step clinical trial.

Although the efficacy of our peptide vaccine was not clearly demonstrated in this phase II study, the timing of and combination treatment with vaccination might not be optimized, and the sample size was limited. Recently, regulatory T cells (Tregs) and myeloid-derived suppressor cells (MDSCs) are reported as potent immunosuppressive cells to protect cancer cells from the host immune system [36,37]. Over expression of PD-L1and PD-1 as well as up-regulation of indoleamine-2,3-dioxygenase (IDO) in the tumor microenvironment also inhibit the CTL functions [38]. Hence, to overcome these immune-escape mechanisms, various approaches have been taken in the last decade [39,40]. For example, anti-PD1antibody [41], anti-PD-L1antibody [42], and anti-CTL4 antibody [43] were applied in clinical trials to overcome the suppressive immuno checkpoints, and surprisingly high objective response rates were observed in many types of malignant neoplasm. Small-molecule inhibitors [44] that block IDO enzymatic activity or cyclophosphamide to reduce the number of Tregs [45] were also applied in clinical trials to dissolve the suppressive immunity. For the successful next generation immunotherapy, peptide vaccine should be combined with some agents to modify the immune-suppressive tumor microenvironments.

In conclusion, our cocktail of five therapeutic epitope peptides appears to be effective in a subset of patients, and warrants a randomized phase III study. In the phase III study, biomarkers such as NLR and lymphocyte-% might be useful for assessing the response to the peptide vaccine and for selecting patients likely to have a better treatment outcome with the vaccination.

## Conclusions

This phase II cancer vaccine therapy demonstrated that our therapeutic peptides cocktail was likely to be effective in a subset of patients and warrants a randomized phase III study. In the phase III study, predictive biomarkers such as NLR and lymphocyte-% should be used for its response and for selecting patients to have a better treatment outcome with the vaccination.

## Abbreviations

RNF43: Ring finger protein 43; TOMM34: 34 kDa-translocase of the outer mitochondrial membrane; KOC1: insulin-like growth factor–II mRNA binding protein 3; VEGFR: Vascular endothelial growth factor receptor; HPLC: High performance liquid chromatography; CRC: Colorectal cancer; ELISPOT: Enzyme-linked immunospot; PBMC: Peripheral blood mononuclear cells; CTL: Cytotoxic T lymphocytes; RR: Response rates; CR: Complete clinical response; SD: Stable disease; PD: Progressive disease; PFS: Progression free survival; OS: Overall survival; HLA: Human leukocyte antigen; MST: Median overall survival time; ECOG: Eastern cooperative oncology group; RECIST: Response evaluation criteria in solid tumors; TIL: Tumor infiltrating cells; CTCAE: Common terminology criteria for adverse events version3.0; AEs: Adverse events; SAEs: Serious adverse events; DLTT: Drug-induced lymphocyte transformation test; PS: Performance status; IFA: Incomplete freund’s adjuvant; CT: Computed tomography; MRI: Magnetic resonance imaging; NLR: Neutrophil/lymphocyte ratio; FOLFOX: Infusional fluorouracil, leucovorin, and oxaliplatin; XELOX: Capecitabine and oxaliplatin, Tregs, regulatory T cells; MDSCs: Myeloid-derived suppressor cells; IDO: Indoleamine-2,3-dioxygenase.

## Competing interests

Yusuke Nakamura is a stock holder and a scientific advisor of OncoTherapy Science, Inc. The other authors have no potential conflicts of interest to disclose.

## Authors’ contributions

SH designed, performed and evaluated clinical study, and wrote the manuscript. YN and MO participated in the design, review and revision of the manuscript. HT, KH, KT, RS, HO, RE, FS, KO, TF, TN, KS, KY, YI, SK, YS, NS, SY, HS, AK, TF, YK and HF assisted to perform clinical study. RT, HT, and TY contributed in the data collection and statistical analysis. All authors participated in the data acquisition and discussion of the manuscript and approved the final manuscript.

## Supplementary Material

Additional file 1 Summary of the protocol. Click here for file
