# Supplementary material for: A phase ΙI study of five peptides combination with oxaliplatin-based chemotherapy as a first-line therapy for advanced colorectal cancer (FXV study)
Source: J Transl Med. 2014 Apr 30;12:108. doi: 10.1186/1479-5876-12-108 (PMC4021539; doi:10.1186/1479-5876-12-108)
Supplement: Additional file 1 — Summary of the protocol. [file 1479-5876-12-108-S1.pdf]

Multicenter Phase II Study of Combination Epitope Peptides with mFOLFOX6 in Advanced/metastatic Colorectal Cancer (ver. 1.0)

A HLA-A-Status Double-Blind, Biologically-Randomized Phase II Study of Five Therapeutic Epitope-Peptides with Oxaliplatin-Based Chemotherapy as First-Line Therapy for Advanced Colorectal Cancer (ver. 1.1)

mFOLFOX6 and Vaccination Study
(FXV study)

Summary of the protocol

Hazama S, et al.

1. Selection of patients

1.1 Inclusion criteria

1) Histologically proven advanced/metastatic unresectable colorectal carcinoma.
2) ECOG Performance Status (PS) of 0 or 1.
3) Patients with measurable one or more Response Evaluation Criteria in Solid Tumors version 1.0 (RECIST) lesions must undergo diagnostic imaging tests within 28 days before registration.
4) Patients with no previous treatment (radiotherapy, chemotherapy) for colorectal cancer, except resection. Patients that have undergone postoperative adjuvant chemotherapy by fluorouracil may be enrolled if relapse is diagnosed beyond week 5 after the final administration.
5) Age: 20 years old or more at registration.
6) Good function of critical organs as defined below.

White blood cell	Ó4000/mm^3 and Ò12000/mm^3
Platelet count	Ó100000/mm^3
Total bilirubin	Ò2.0 mg/dL
AST and ALT	Òless than 2.5-times the upper limit of normal at each sites
Serum creatinine	Ò1.2 mg/dL

7) Expect for more than 3 months alive.
8) Voluntarily signed the written consent form.

1.2 Exclusion criteria

1) Serious complications (e.g. heart failure, hemorrhagic peptic ulcer).
2) Pregnant females, possibly pregnant females, females wishing to become pregnant and nursing mothers. Males that are currently attempting to produce a pregnancy.
3) Active infection beyond control
4) Steroid or immunosuppressive therapy
5) Allergy for using epitope peptides
6) Mental and nervous disorder including metastasis in the CNS or severe mental disorder.
7) Massive ascites or pleural effusion requiring treatment.
8) Peripheral nervous disorder
9) Watery diarrhea.
10) Pulmonary fibrosis or interstitial pneumonia
11) Active double cancer (synchronous double cancer or asynchronous double cancer with disease-free duration of 3 years or less).

12) Judged ineligible by physicians for participation in the study.

2. Study Schema

Open-labeled, single armed, multi-center (13), biologically-randomized, Phase II comparative study as an exploratory setting.

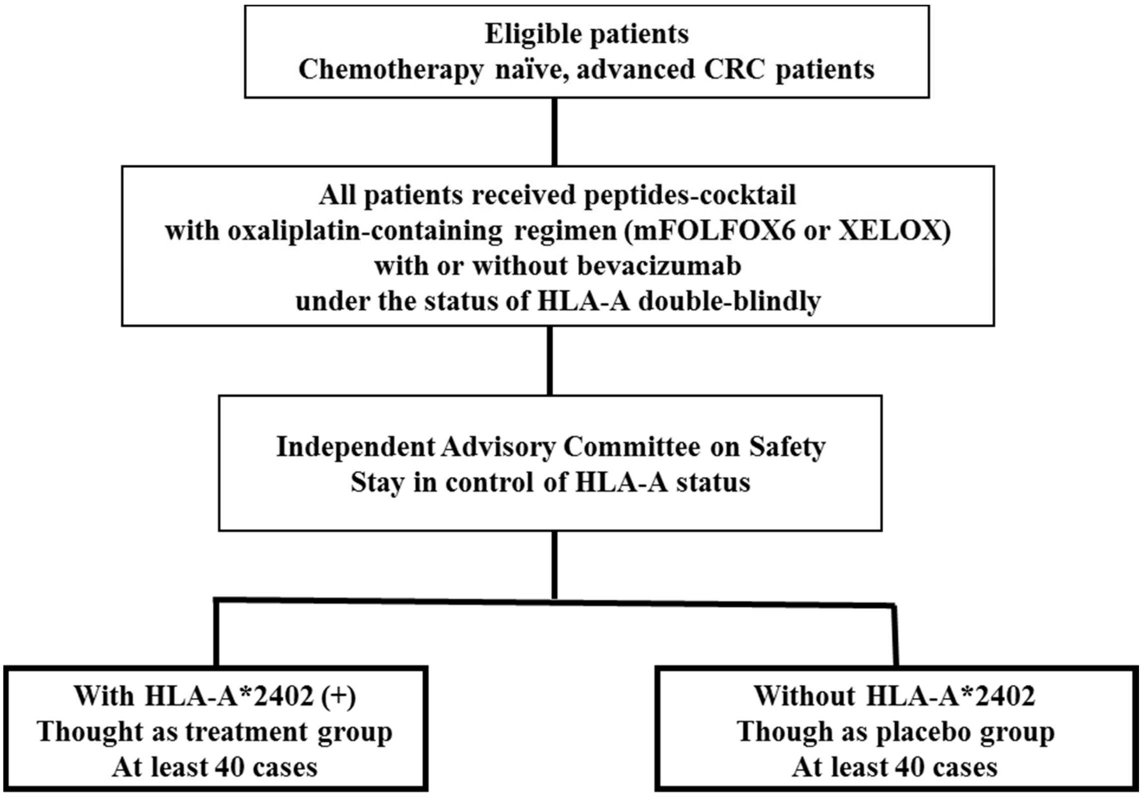

The Data and Safety Monitoring Committee of this study will monitor the HLA-A status of every patient as double blindly for both patients and physicians. The Committee also will advise, and make a designation to the principal investigator for stopping patientsô enrollment when both arms may be reached 40 patients.

3. Treatment plan and rules for dose modification

3.0 Treatment regimen

Although the peptides used in this study will be HLA-A*2402 restricted peptides, all enrolled patients who will be double-blinded for HLA-A status will be administrated the same regimen of peptide cocktail and oxaliplatin-containing chemotherapy.

3.1 Dosage, mode of administration and dosage schedule

1) Peptide vaccine.

All patients will receive peptide cocktail. The cocktail of 5 peptides derived from RNF43-721, TOMM34-299, KOC1-508, VEGFR1-1084 and VEGFR2-169 at the dose of 3 mg will be mixed with 1.5 ml of incomplete Freund's adjuvant (IFA) (Montanide ISA51; Seppic, Paris, France) will be administered subcutaneously into the thigh or axilla regions on day 1 of every week, and then after thirteen weeks, the vaccination schedule will be reduced to be biweekly. Vaccination may be continued even after the progression of the disease when a patient may wish and a primary doctor, who provides additional chemotherapies, may recommend.

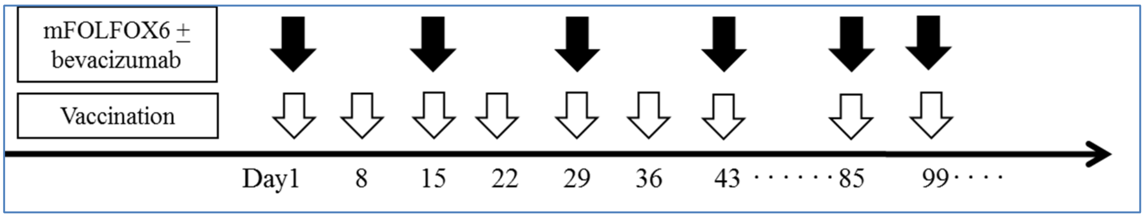

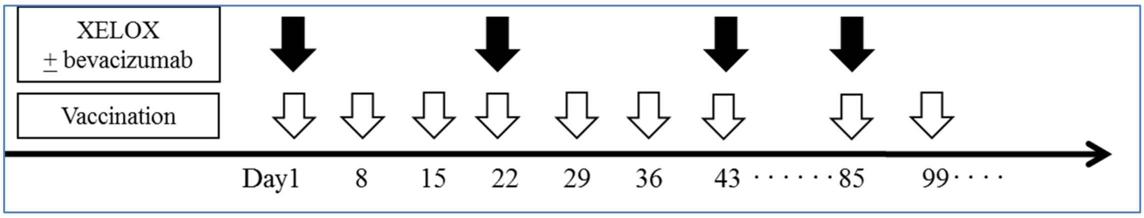

2) Chemotherapy

All patients will receive modified FOLFOX6 or XELOX, and with/without bevacizumab, in addition to the peptide cocktail. The investigator will select which of these chemotherapies the patient will receive. The choice of chemotherapy (FOLFOX or XELOX, and with/without bevacizumab) will not be randomized. mFOLFOX6 or XELOX will be administered according to the general clinical practice.

2-1) Modified FOLFOX6 with/without bevacizumab

mFOLFOX6 will be administered according to the general clinical practice. mFOLFOX6 is consisted of intravenous infusion of oxaliplatin 85 mg/m^2 on day 1 with leucovorin 400mg/m^2, followed by FU 400 mg/m^2 bolus, and then 2,400 mg/m^2 continuous infusion over 46 hours with/without bevacizumab 5 mg/kg intravenous infusion before oxaliplatin every 14 days, as described previously.[1,2]

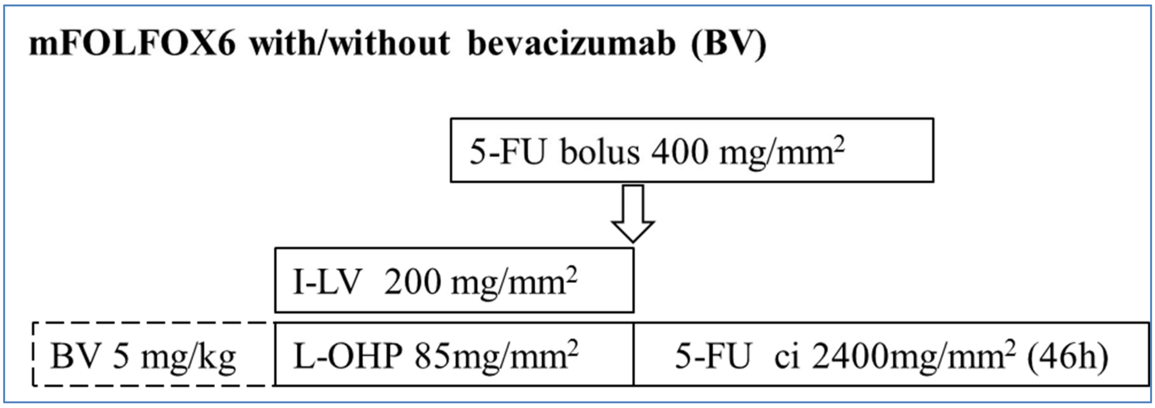

2-2) XELOX with/without bevacizumab

XELOX will be administered according to the general clinical practice. XELOX is consisted of intravenous infusion of oxaliplatin 130 mg/m^2 on day 1 followed by oral capecitabine 1,000 mg/m^2 twice daily on days 1 through 14 of a 21-day cycle with/without bevacizumab infusion before oxaliplatin at a dose of 7.5 mg/kg, as described previously.[2]

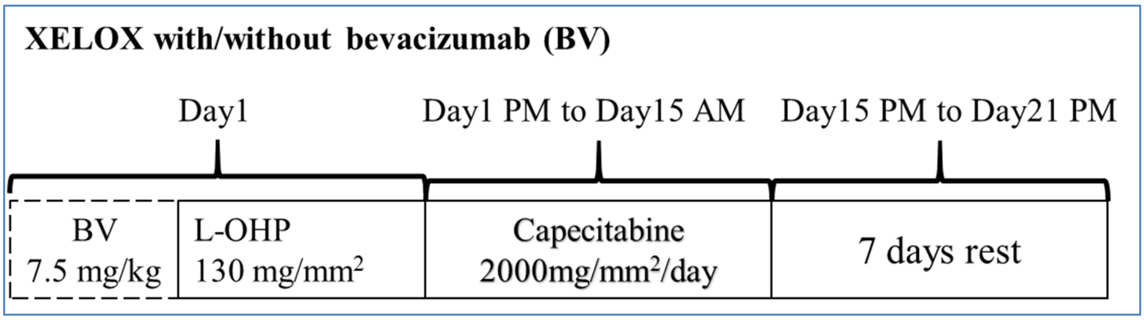

3.2 Criteria for the start of each cycle

1) Peptide vaccine

1. No Grade 3 or more injection site toxicity which may be decided as discontinuation of administration.
2. No decisions as intolerable to continue the injection of peptide vaccine for any reason by physicians for participation in the study.

2) mFOLFOX6 and XELOX

mFOLFOX6 or XELOX will be administered according to the general clinical practice. After confirmation that each subject satisfies criteria for the start of each cycle described in Table below, mFOLFOX6 or XELOX will be administered. If any criteria are not satisfied, administration of the cycle will be postponed. After confirmation that the criteria described in Table below are satisfied, administration will be initiated. From the 2nd cycle and thereafter, the study treatment will be discontinued if administration of the cycle has not been initiated by 14 days after the estimated day of administration. If the patient may not meet the retreatment criteria in this timeframe, he/she will be taken off protocol treatment.

Criteria for start of cycle (criteria for administration on Day 1 of each cycle)

Parameter		Criterion for start
White blood cell count		Ó3,000/mm^3
Neutrophil count		Ó1,500/mm^3
Platelet count		Ó75,000/mm^3
Infection		No fever（more than38.0℃）suspect for infection
Non-hematologic toxicities	diarrhea	No watery diarrhea
	others	Ó Grade2 (CTCAE grading, except for nausea, vomiting, anorexia, and fatigue)

3) Bevacizumab

Bevacizumab will be administered according to the general clinical practice. If the chemotherapy (FOLFOX or XELOX) may be held or delayed for toxicity reasons, bevacizumab will be to be delayed as well and was to be resumed with the chemotherapy. This applied even if the toxicity may not relate to bevacizumab. Any patient who may develop any one of the following toxicities will be not to receive further bevacizumab: 1) Gastrointestinal perforation, 2) Arterial thromboembolic events, 3) Grade 3/4 hemorrhagic events, 4) Symptomatic Grade 4 venous thromboembolic

events, 5) Grade 4 hypertension (hypertensive crisis), 6) Grade 4 proteinuria (nephrotic syndrome)

3.3 Criteria for dose reduction

1) Peptide vaccine

The dosage of peptide vaccine will not be modified.

2) mFOLFOX6

(1) Table below provides guidance for dose reductions for the first appearance of the specified toxicities. Based on the most severe toxicity experienced since the last treatment, the following dose modifications will be used for non-hematological toxicities. When the dose of 5-FU may be reduced, the dose of leucovorin will be to remain the same.

Criteria for dose reductions for the first appearance of the specified toxicities.

<table>
<tr><th>Criteria</th><th>L-OHP</th><th>5-FU (bolus)</th><th>5-FU (continuous)</th></tr>
<tr><td>ÓGrade 3, leukopenia, or neutropenia
ÓGrade 2, thrombocytopenia</td><td rowspan="2">20% dose reduction</td><td rowspan="2">Stop treatment</td><td rowspan="2">No dose reduction</td></tr>
<tr><td>ÓGrade 3, non-hematological toxicity</td></tr>
<tr><td>Grade 2, neurologic toxicities</td><td>20% dose reduction</td><td rowspan="2">No dose reduction</td><td rowspan="2">No dose reduction</td></tr>
<tr><td>Grade 3, neurologic toxicities</td><td>Stop treatment</td></tr>
<tr><td>Allergic reactions or Respiratory symptoms indicative of pulmonary fibrosis due to L-OHP</td><td>Stop treatment</td><td>No dose reduction</td><td>No dose reduction</td></tr>
</table>

Note: the dose of leucovorin remained the same.

(2) Table below provides guidance for dose reductions for the second appearance of the specified toxicities. Based on the most severe toxicity experienced since the last treatment, the following dose modifications will be used for non-hematological toxicities. When the dose of 5-FU may be reduced, the dose of leucovorin will be to remain the same.

<table>
<tr><th>Criteria</th><th>L-OHP</th><th>5-FU (bolus)</th><th>5-FU (continuous)</th></tr>
<tr><td>ÓGrade 3, leukopenia, or neutropenia
ÓGrade 2, thrombocytopenia</td><td rowspan="2">20% dose reduction</td><td rowspan="2">-</td><td rowspan="2">20% dose reduction</td></tr>
<tr><td>ÓGrade 3, non-hematological toxicity</td></tr>
<tr><td>Grade 2, neurologic toxicities</td><td>20% dose reduction</td><td rowspan="2">No dose reduction</td><td rowspan="2">No dose reduction</td></tr>
<tr><td>Grade 3, neurologic toxicities</td><td>Stop treatment</td></tr>
<tr><td>Allergic reactions or Respiratory symptoms indicative of pulmonary fibrosis due to L-OHP</td><td>Stop treatment</td><td>No dose reduction</td><td>No dose reduction</td></tr>
</table>

Note: the dose of leucovorin remained the same.

3.4 Criteria for discontinuation of study treatment

When the following criteria will be met in patients, the protocol treatment will be stopped according to the attending physiciansôdecision.

1) Obvious progressive disease according to RECIST. (Vaccination may be continued even after the progression of the disease when a patient may wish and a primary doctor, who provides additional chemotherapies, may recommend.)
2) If the tumors become resectable resulting from the tumor shrinkage.
3) If the treatment is delayed more than 15 days after the next scheduled cycle of treatment.
4) If an adverse event satisfying the criteria for dose reduction occurs even after reduction to the lowest level.
5) If an adverse event that prevents continuation of the study treatment occurs.
6) If attending physicians judge that the clinical trial should be discontinued due to aggravation of a pathological condition or complications.
7) If the subject requests discontinuation of administration.
8) If attending physicians judge that discontinuation of administration is necessary.

4. Efficacy parameters

4.0 Assessments

Medical history, physical examination, chest X-ray, ECG, and carcinoembryonic antigen (CEA) measurements were performed within 21 days before starting the treatment. Assessments of vital signs, ECOG performance status, height, weight, and routine blood analysis (hematology and chemistry) were performed within 7 days of starting the treatment. During treatment, physical examination, hematology, and biochemistry analyses were repeated on day 1 of every treatment cycle. Tumor assessments (computed tomography scan, magnetic resonance imaging) were made before starting the study treatment and were repeated every 4 to 8 weeks after the treatment. RECIST guidelines were used to define all responses. Signs of hematological toxicity and non-hematological toxicity were assessed according to CTCAE during therapy and for 28 days after the last study drug dose.

4.1 Overall survival

Overall survival will be measured from the first day of protocol treatment until the day of patient death (from any cause). For the survival cases at final follow-up examination, evaluation will end on the day of final confirmation of survival.

4.2 Progression

òProgressionò as an event during progression-free survival (PFS) includes both progressive disease (PD) confirmed on images specified by RECIST and progression of primary diseases not specified by RECIST, plus appearance of new lesions (apparent progression based on clinical diagnosis). If progression may be first diagnosed by aggravation of clinical symptoms, the day in which progression has been confirmed will be the day of judgment of progression, even if imaging is conducted later.

4.3 Judgment of tumor reducing effect

Of all patient registered, those with measurable lesions satisfying the items described below will be evaluated for tumor reducing effect based on RECIST criteria to calculate response rate.

5. Endpoints

5.1 Primary endpoint

5.1.1 Response rate (RR)

Response rate is defined as the ratio of subjects that achieved complete response (CR) or partial response (PR) (best overall response) in subjects with measurable lesions specified by RECIST.

5.1.2 Progression-free survival (PFS)

PFS is defined as the period from the first day of protocol treatment to the day of diagnosis of tumor progression or to patient death (from any cause) if the patient died without diagnosis of tumor progression. For surviving patients with no tumor progression, evaluation will be ended on the day of final confirmation of progression-free survival. If the patients may receive surgical resection, evaluation will be ended on the day of operation.

5.2 Secondary endpoint

5.2.1 Overall survival (OS)

Overall survival (OS) is defined as the period from the first day of protocol treatment to death from any cause. For survival cases, evaluation will be ended on the day of final confirmation of survival. If the patients may receive surgical resection, their survival time will be regarded as the period from the first day of protocol treatment to the day of operation.

5.2.2 Safety

For adverse events and adverse drug reactions specified in the protocol, incidence rates of adverse events marking the worst grade during the study period will be calculated using CTCAE v3.0.

5.2.3 Disease control rate

Disease control rate is defined as the ratio of subjects that achieved CR or PR or stable disease (SD) (best overall response) in subjects with measurable lesions specified by RECIST.

5.2.4 Antigen-specific immune responses

Antigen-specific T cell response will be estimated by ELISPOT assay following in vitro sensitization. Antigen specific T cell response will be classified into four grades (-, +,

++, or +++) according to the algorithm flow chart described in our previous report[3] (+++: IFN-γ producing cell is contained more than 0.2 %, ++ : IFN-γ producing cell is contained 0.02 - 0.2 %, + : IFN-γ producing cell is contained 0.01 - 0.02 %, ï : IFN-γ producing cell is contained less than 0.01% in the sample applied for ELISPOT). Sensitivity of our ELISPOT assay was estimated at approximately average level by the ELISPOT panel of the Cancer Immunotherapy Consortium.[4]

6. Statistical analysis

This study is designed to test the hypothesis that a regime consisting of vaccination plus oxaliplatin-containing chemotherapy is an effective treatment for patients with HLA-A*2402 positive advanced colorectal carcinoma when compared to patients without HLA-A*2402. Because the response rate of colorectal cancer patients to first line-treatment is generally about 50%, we estimate that a minimum of 40 patients for both arms will be required, assuming a response rate of 50% in the HLA-A*2402 negative group and 65% in the A*2402 positive group. Alpha levels of 0.2 and beta levels of 0.5 are assumed as the exploratory settings. The Data and Safety Monitoring Committee of this study monitored the HLA-A status of every patient using a double blind for both patients and physicians. The Committee also will advise the principal investigator to stop patient enrollment when both arms may reach 40 patients.

OS rates and PFS rates will be analyzed by the Kaplan-Meier method. Survival will be measured in days from the first vaccination to succumbing to the disease. Patients who may have curative resection of the tumor will be handled as censored cases as of the operation day. P-values will be assessed using a log-rank test.

REFERENCES

1. **Kato T, Muro K, Yamaguchi K, et al: Cediranib in combination with mFOLFOX6 in Japanese patients with metastatic colorectal cancer: results from the randomised phase II part of a phase I/II study. Ann Oncol 23:933-41, 2012**
2. **Cassidy J, Clarke S, Diaz-Rubio E, et al: Randomized phase III study of capecitabine plus oxaliplatin compared with fluorouracil/folinic acid plus oxaliplatin as first-line therapy for metastatic colorectal cancer. J Clin Oncol 26:2006-12, 2008**
3. **Suzuki N, Hazama S, Ueno T, et al: A Phase I Clinical Trial of Vaccination With**

KIF20A-derived Peptide in Combination With Gemcitabine For Patients With Advanced Pancreatic Cancer. J Immunother 37:36-42, 2014

4. Janetzki S, Panageas KS, Ben-Porat L, et al: Results and harmonization guidelines from two large-scale international Elispot proficiency panels conducted by the Cancer Vaccine Consortium (CVC/SVI). Cancer Immunol Immunother 57:303-15, 2008
